# (4,6-Diamino-1,3-phenyl­ene)bis­(phenyl­methanone)

**DOI:** 10.1107/S1600536811032752

**Published:** 2011-09-14

**Authors:** Da-Min Tian, Hui Xu

**Affiliations:** a Department of Chemistry and Chemical Engineering, Henan University of Urban Construction, Henan 467036, People’s Republic of China

## Abstract

In the mol­ecule of the title compound, C_20_H_16_N_2_O_2_, two intra­molecular N—H⋯O inter­actions occur. The mol­ecular chains are linked by N—H⋯π and C—H⋯π inter­actions into a three-dimensional network, which seems to be very effective in the stabilization of the crystal structure.

## Related literature

For background on the applications of the title compound, see: Imai *et al.* (1975 ▶). For the synthetic procedure for the title compound, see: Zhang *et al.* (2011 ▶). For bond-length data, see: Allen *et al.* (1987 ▶).
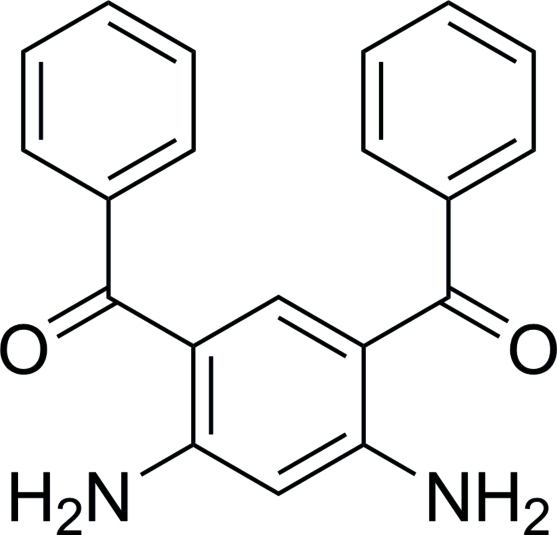


## Experimental

### 

#### Crystal data

C_20_H_16_N_2_O_2_
*M* *_r_* = 316.35
Monoclinic, 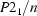

*a* = 13.602 (3) Å
*b* = 7.2350 (14) Å
*c* = 16.786 (3) Å
β = 104.32 (3)°
*V* = 1600.6 (5) Å^3^
*Z* = 4
Mo *K*α radiation
μ = 0.09 mm^−1^
*T* = 293 K
0.30 × 0.20 × 0.10 mm

#### Data collection

Nonius CAD-4 diffractometer
Absorption correction: ψ scan (North *et al.*, 1968 ▶) *T*_min_ = 0.975, *T*_max_ = 0.992
3034 measured reflections
2905 independent reflections
1956 reflections with *I* > 2σ(*I*)
*R*_int_ = 0.050
3 standard reflections every 200 reflections  intensity decay: 1%

#### Refinement

*R*[*F* ^2^ > 2σ(*F* ^2^)] = 0.052
*wR*(*F* ^2^) = 0.159
*S* = 1.00
2905 reflections
218 parameters
H-atom parameters constrained
Δρ_max_ = 0.21 e Å^−3^
Δρ_min_ = −0.16 e Å^−3^

### 

Data collection: *CAD-4 EXPRESS* (Enraf–Nonius, 1985 ▶); cell refinement: *CAD-4 EXPRESS*; data reduction: *XCAD4* (Harms & Wocadlo, 1995 ▶); program(s) used to solve structure: *SHELXS97* (Sheldrick, 2008 ▶); program(s) used to refine structure: *SHELXL97* (Sheldrick, 2008 ▶); molecular graphics: *SHELXTL* (Sheldrick, 2008 ▶); software used to prepare material for publication: *SHELXTL*.

## Supplementary Material

Crystal structure: contains datablock(s) I, TDM. DOI: 10.1107/S1600536811032752/vm2115sup1.cif

Structure factors: contains datablock(s) I. DOI: 10.1107/S1600536811032752/vm2115Isup2.hkl

Supplementary material file. DOI: 10.1107/S1600536811032752/vm2115Isup3.cml

Additional supplementary materials:  crystallographic information; 3D view; checkCIF report

## Figures and Tables

**Table 1:** Hydrogen-bond geometry (Å, °) *Cg*1 and *Cg*2 are the centroids of the C1–C6 and C8–C13, rings respectively.

*D*—H⋯*A*	*D*—H	H⋯*A*	*D*⋯*A*	*D*—H⋯*A*
N1—H1*B*⋯O1	0.86	2.04	2.676 (3)	130
N2—H2*B*⋯O2	0.86	2.05	2.688 (3)	131
C19—H19*A*⋯*Cg*1^i^	0.93	2.79	3.555 (3)	140
N1—H1*A*⋯*Cg*2^ii^	0.86	2.71	3.550 (2)	167

Symmetry codes: (i) 
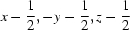
; (ii) 
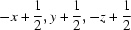
.

**Fig. 1.**
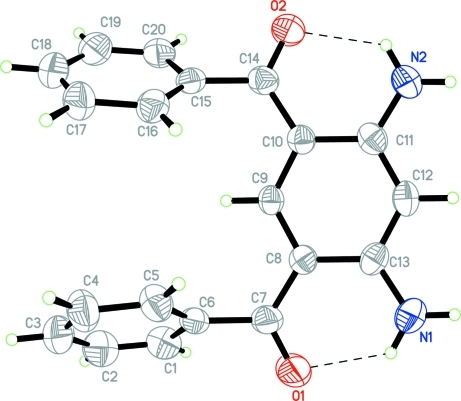
The molecular structure of (I), with the atom-numbering scheme. Displacement ellipsoids are drawn at the 50% probability level. Hydrogen bonds are shown as dashed lines.

## supplementary crystallographic information

### Comment

The title compound, (4,6-diamino-1,3-phenylene)bis(phenylmethanone) is an important intermediate, which can be utilized to synthesize organic semiconductors and conjugated polymers (Imai *et al.*, 1975). We report here its crystal structure (Fig. 1).

There are two intramolecular N—H···O interactions (Table 1) in one molecule. The bond lengths and angles are within normal ranges (Allen *et al.*, 1987).

In the molecule of the title compound, the rings are planar. The dihedral angles of the rings A(C1—C6), B(C8—C13), C(C15—C20) are: A/B = 61.1 (1)°, A/C = 66.6 (2)°, B/C = 55.3 (1)°.

In the crystal structure, the molecular chains are linked by N—H···π  and C—H···π interactions into a three-dimensional network, which seems to be very effective in the stabilization of the crystal structure [C19—H19A···*Cg*1^i^ 2.79Å, N1—H1A···*Cg*2^ii^ 2.71 Å (*Cg*1 and *Cg*2 are the centroids of the rings defined by the atoms C1—C6 and C8—C13, respectively; symmetry codes: (i) -1/2 + *x*, -1/2 - *y*, -1/2 + *z*, (ii) 1/2 - *x*, 1/2 + *y*, 1/2 - *z*)].

The molecular symmetry is best described by point group *C_s_*. In the crystal structure, molecules are stacked parallel to the *a* axis direction.

### Experimental

The title compound was prepared by the method of Ullmann reaction reported in literature (Zhang *et al.*, 2011). Crystals were obtained by dissolving the produc t (0.2 g, 0.63 mmol) in ethanol (25 ml) and evaporating the solvent slowly at room temperature for about 5 d.

### Refinement

The H atoms of the amino groups were located in a difference Fourier map, and refined with a distance restraint N—H = 0.86 Å. Other H atoms were positioned geometrically and refined as riding groups, with C—H = 0.93 Å for aromatic H, and constrained to ride on their parent atoms, with *U*_iso_(H) = *xU*_eq_(C), where *x* = 1.2 for aromatic H, and *x* = 1.5 for other H.

### Crystal data

**Table:**

C_20_H_16_N_2_O_2_	*F*(000) = 664
*M**_r_* = 316.35	*D*_x_ = 1.313 Mg m^−^^3^
Monoclinic, *P*2_1_/*n*	Mo *K*α radiation, λ = 0.71073 Å
Hall symbol: -P 2yn	Cell parameters from 25 reflections
*a* = 13.602 (3) Å	θ = 9–13°
*b* = 7.2350 (14) Å	µ = 0.09 mm^−^^1^
*c* = 16.786 (3) Å	*T* = 293 K
β = 104.32 (3)°	Block, colourless
*V* = 1600.6 (5) Å^3^	0.30 × 0.20 × 0.10 mm
*Z* = 4	

### Data collection

**Table:**

Nonius CAD-4 diffractometer	1956 reflections with *I* > 2σ(*I*)
Radiation source: fine-focus sealed tube	*R*_int_ = 0.050
graphite	θ_max_ = 25.3°, θ_min_ = 1.7°
ω/2θ scans	*h* = 0→16
Absorption correction: ψ scan (North *et al.*, 1968)	*k* = 0→8
*T*_min_ = 0.975, *T*_max_ = 0.992	*l* = −20→19
3034 measured reflections	3 standard reflections every 200 reflections
2905 independent reflections	intensity decay: 1%

### Refinement

**Table:**

Refinement on *F*^2^	Secondary atom site location: difference Fourier map
Least-squares matrix: full	Hydrogen site location: inferred from neighbouring sites
*R*[*F*^2^ > 2σ(*F*^2^)] = 0.052	H-atom parameters constrained
*wR*(*F*^2^) = 0.159	*w* = 1/[σ^2^(*F*_o_^2^) + (0.095*P*)^2^] where *P* = (*F*_o_^2^ + 2*F*_c_^2^)/3
*S* = 1.00	(Δ/σ)_max_ < 0.001
2905 reflections	Δρ_max_ = 0.21 e Å^−^^3^
218 parameters	Δρ_min_ = −0.16 e Å^−^^3^
0 restraints	Extinction correction: *SHELXL*, Fc^*^=kFc[1+0.001xFc^2^λ^3^/sin(2θ)]^-1/4^
Primary atom site location: structure-invariant direct methods	Extinction coefficient: 0.033 (4)

### Special details

**Table:**

Geometry. All e.s.d.'s (except the e.s.d. in the dihedral angle between two l.s. planes) are estimated using the full covariance matrix. The cell e.s.d.'s are taken into account individually in the estimation of e.s.d.'s in distances, angles and torsion angles; correlations between e.s.d.'s in cell parameters are only used when they are defined by crystal symmetry. An approximate (isotropic) treatment of cell e.s.d.'s is used for estimating e.s.d.'s involving l.s. planes.
Refinement. Refinement of *F*^2^ against ALL reflections. The weighted *R*-factor *wR* and goodness of fit *S* are based on *F*^2^, conventional *R*-factors *R* are based on *F*, with *F* set to zero for negative *F*^2^. The threshold expression of *F*^2^ > σ(*F*^2^) is used only for calculating *R*-factors(gt) *etc*. and is not relevant to the choice of reflections for refinement. *R*-factors based on *F*^2^ are statistically about twice as large as those based on *F*, and *R*- factors based on ALL data will be even larger.

### Fractional atomic coordinates and isotropic or equivalent isotropic displacement parameters (Å^2^)

**Table:**

	*x*	*y*	*z*	*U*_iso_*/*U*_eq_	
N1	0.37312 (16)	0.3893 (3)	0.28367 (12)	0.0591 (6)	
H1A	0.3491	0.4712	0.3109	0.071*	
H1B	0.4341	0.3493	0.3021	0.071*	
O1	0.52000 (14)	0.1831 (3)	0.24813 (14)	0.0930 (8)	
C1	0.57390 (17)	−0.0458 (4)	0.12198 (14)	0.0531 (6)	
H1C	0.6095	0.0647	0.1245	0.064*	
O2	0.04343 (12)	0.1873 (2)	−0.05001 (10)	0.0611 (5)	
N2	0.05679 (14)	0.3923 (3)	0.08577 (13)	0.0578 (6)	
H2A	0.0370	0.4769	0.1142	0.069*	
H2B	0.0163	0.3519	0.0415	0.069*	
C2	0.60783 (19)	−0.2017 (4)	0.08967 (15)	0.0607 (7)	
H2C	0.6656	−0.1947	0.0696	0.073*	
C3	0.5578 (2)	−0.3659 (4)	0.08670 (16)	0.0604 (7)	
H3A	0.5818	−0.4704	0.0653	0.072*	
C4	0.4720 (2)	−0.3763 (3)	0.11544 (16)	0.0589 (7)	
H4A	0.4378	−0.4881	0.1138	0.071*	
C5	0.43617 (17)	−0.2205 (3)	0.14694 (14)	0.0477 (6)	
H5A	0.3775	−0.2280	0.1658	0.057*	
C6	0.48687 (15)	−0.0536 (3)	0.15059 (12)	0.0382 (5)	
C7	0.45547 (17)	0.1125 (3)	0.19187 (14)	0.0468 (6)	
C8	0.35224 (15)	0.1836 (3)	0.16598 (12)	0.0348 (5)	
C9	0.28558 (14)	0.1203 (3)	0.09414 (12)	0.0333 (5)	
H9A	0.3095	0.0308	0.0639	0.040*	
C10	0.18605 (15)	0.1806 (3)	0.06417 (12)	0.0348 (5)	
C11	0.15099 (16)	0.3230 (3)	0.11055 (13)	0.0399 (5)	
C12	0.21684 (16)	0.3878 (3)	0.18220 (13)	0.0431 (6)	
H12A	0.1936	0.4795	0.2118	0.052*	
C13	0.31502 (16)	0.3235 (3)	0.21194 (12)	0.0395 (5)	
C14	0.12288 (16)	0.1100 (3)	−0.01357 (13)	0.0402 (5)	
C15	0.15422 (15)	−0.0590 (3)	−0.05239 (12)	0.0384 (5)	
C16	0.18537 (17)	−0.2215 (3)	−0.00953 (14)	0.0443 (6)	
H16A	0.1921	−0.2251	0.0469	0.053*	
C17	0.20657 (18)	−0.3783 (3)	−0.04972 (17)	0.0562 (7)	
H17A	0.2271	−0.4864	−0.0203	0.067*	
C18	0.19713 (19)	−0.3735 (4)	−0.13331 (18)	0.0642 (8)	
H18A	0.2113	−0.4785	−0.1604	0.077*	
C19	0.1668 (2)	−0.2136 (4)	−0.17665 (16)	0.0621 (7)	
H19A	0.1614	−0.2104	−0.2330	0.075*	
C20	0.14434 (17)	−0.0577 (4)	−0.13704 (14)	0.0523 (6)	
H20A	0.1224	0.0489	−0.1671	0.063*	

### Atomic displacement parameters (Å^2^)

**Table:**

	*U*^11^	*U*^22^	*U*^33^	*U*^12^	*U*^13^	*U*^23^
N1	0.0621 (13)	0.0564 (13)	0.0529 (12)	0.0047 (11)	0.0032 (10)	−0.0213 (10)
O1	0.0592 (12)	0.0820 (15)	0.1104 (16)	0.0206 (11)	−0.0310 (11)	−0.0536 (13)
C1	0.0470 (14)	0.0519 (16)	0.0608 (15)	−0.0068 (12)	0.0144 (11)	0.0068 (12)
O2	0.0525 (10)	0.0619 (11)	0.0576 (10)	0.0207 (9)	−0.0077 (8)	−0.0073 (9)
N2	0.0437 (11)	0.0595 (14)	0.0667 (13)	0.0159 (10)	0.0072 (10)	−0.0157 (11)
C2	0.0526 (15)	0.079 (2)	0.0580 (16)	0.0050 (14)	0.0274 (13)	−0.0004 (14)
C3	0.0603 (16)	0.0593 (17)	0.0626 (16)	0.0119 (14)	0.0172 (13)	−0.0141 (14)
C4	0.0583 (15)	0.0388 (14)	0.0792 (18)	0.0033 (12)	0.0162 (13)	−0.0054 (13)
C5	0.0433 (13)	0.0426 (14)	0.0596 (15)	0.0017 (10)	0.0171 (11)	−0.0003 (11)
C6	0.0347 (11)	0.0375 (12)	0.0386 (11)	0.0022 (9)	0.0021 (9)	0.0016 (10)
C7	0.0441 (13)	0.0389 (13)	0.0502 (13)	0.0016 (11)	−0.0020 (10)	−0.0034 (11)
C8	0.0399 (11)	0.0267 (11)	0.0376 (11)	−0.0002 (9)	0.0089 (9)	0.0023 (9)
C9	0.0384 (11)	0.0262 (10)	0.0361 (11)	0.0014 (9)	0.0107 (9)	0.0006 (8)
C10	0.0380 (11)	0.0299 (11)	0.0363 (11)	0.0027 (9)	0.0090 (9)	0.0034 (9)
C11	0.0405 (12)	0.0320 (12)	0.0484 (13)	0.0026 (10)	0.0134 (10)	0.0039 (10)
C12	0.0508 (13)	0.0341 (12)	0.0478 (13)	0.0048 (10)	0.0184 (10)	−0.0055 (10)
C13	0.0493 (13)	0.0327 (12)	0.0369 (11)	−0.0054 (10)	0.0113 (9)	−0.0038 (9)
C14	0.0384 (12)	0.0375 (12)	0.0426 (12)	0.0024 (10)	0.0057 (9)	0.0041 (10)
C15	0.0323 (11)	0.0369 (12)	0.0422 (12)	−0.0021 (9)	0.0020 (9)	−0.0020 (10)
C16	0.0458 (13)	0.0377 (13)	0.0476 (13)	−0.0031 (10)	0.0082 (10)	−0.0019 (10)
C17	0.0540 (15)	0.0364 (13)	0.0764 (18)	0.0019 (11)	0.0125 (13)	−0.0043 (13)
C18	0.0567 (16)	0.0599 (18)	0.0722 (18)	0.0055 (13)	0.0089 (13)	−0.0270 (15)
C19	0.0599 (16)	0.076 (2)	0.0454 (14)	0.0069 (15)	0.0041 (12)	−0.0158 (14)
C20	0.0482 (13)	0.0583 (16)	0.0442 (13)	0.0086 (12)	−0.0001 (10)	−0.0016 (12)

### Geometric parameters (Å, °)

**Table:**

N1—C13	1.353 (3)	C8—C9	1.395 (3)
N1—H1A	0.8600	C8—C13	1.439 (3)
N1—H1B	0.8600	C9—C10	1.392 (3)
O1—C7	1.230 (3)	C9—H9A	0.9300
C1—C2	1.380 (3)	C10—C11	1.442 (3)
C1—C6	1.384 (3)	C10—C14	1.465 (3)
C1—H1C	0.9300	C11—C12	1.391 (3)
O2—C14	1.236 (2)	C12—C13	1.385 (3)
N2—C11	1.342 (3)	C12—H12A	0.9300
N2—H2A	0.8600	C14—C15	1.496 (3)
N2—H2B	0.8600	C15—C16	1.389 (3)
C2—C3	1.364 (4)	C15—C20	1.394 (3)
C2—H2C	0.9300	C16—C17	1.386 (3)
C3—C4	1.371 (4)	C16—H16A	0.9300
C3—H3A	0.9300	C17—C18	1.378 (4)
C4—C5	1.384 (3)	C17—H17A	0.9300
C4—H4A	0.9300	C18—C19	1.374 (4)
C5—C6	1.384 (3)	C18—H18A	0.9300
C5—H5A	0.9300	C19—C20	1.381 (3)
C6—C7	1.501 (3)	C19—H19A	0.9300
C7—C8	1.457 (3)	C20—H20A	0.9300
			
C13—N1—H1A	120.0	C9—C10—C11	116.78 (18)
C13—N1—H1B	120.0	C9—C10—C14	121.08 (19)
H1A—N1—H1B	120.0	C11—C10—C14	122.04 (18)
C2—C1—C6	120.1 (2)	N2—C11—C12	120.2 (2)
C2—C1—H1C	120.0	N2—C11—C10	121.1 (2)
C6—C1—H1C	120.0	C12—C11—C10	118.65 (19)
C11—N2—H2A	120.0	C13—C12—C11	123.7 (2)
C11—N2—H2B	120.0	C13—C12—H12A	118.1
H2A—N2—H2B	120.0	C11—C12—H12A	118.1
C3—C2—C1	120.9 (2)	N1—C13—C12	120.1 (2)
C3—C2—H2C	119.5	N1—C13—C8	121.3 (2)
C1—C2—H2C	119.5	C12—C13—C8	118.60 (18)
C2—C3—C4	119.7 (2)	O2—C14—C10	122.1 (2)
C2—C3—H3A	120.2	O2—C14—C15	117.56 (18)
C4—C3—H3A	120.2	C10—C14—C15	120.34 (18)
C3—C4—C5	120.1 (2)	C16—C15—C20	118.3 (2)
C3—C4—H4A	120.0	C16—C15—C14	123.33 (19)
C5—C4—H4A	120.0	C20—C15—C14	118.21 (19)
C4—C5—C6	120.6 (2)	C17—C16—C15	121.0 (2)
C4—C5—H5A	119.7	C17—C16—H16A	119.5
C6—C5—H5A	119.7	C15—C16—H16A	119.5
C5—C6—C1	118.7 (2)	C18—C17—C16	119.8 (2)
C5—C6—C7	121.5 (2)	C18—C17—H17A	120.1
C1—C6—C7	119.5 (2)	C16—C17—H17A	120.1
O1—C7—C8	122.3 (2)	C19—C18—C17	120.0 (2)
O1—C7—C6	117.2 (2)	C19—C18—H18A	120.0
C8—C7—C6	120.56 (18)	C17—C18—H18A	120.0
C9—C8—C13	117.07 (18)	C18—C19—C20	120.4 (2)
C9—C8—C7	120.94 (19)	C18—C19—H19A	119.8
C13—C8—C7	121.98 (18)	C20—C19—H19A	119.8
C10—C9—C8	125.14 (19)	C19—C20—C15	120.6 (2)
C10—C9—H9A	117.4	C19—C20—H20A	119.7
C8—C9—H9A	117.4	C15—C20—H20A	119.7
			
C6—C1—C2—C3	−1.2 (4)	N2—C11—C12—C13	179.9 (2)
C1—C2—C3—C4	0.6 (4)	C10—C11—C12—C13	0.1 (3)
C2—C3—C4—C5	0.3 (4)	C11—C12—C13—N1	−177.7 (2)
C3—C4—C5—C6	−0.7 (4)	C11—C12—C13—C8	0.9 (3)
C4—C5—C6—C1	0.1 (3)	C9—C8—C13—N1	177.98 (19)
C4—C5—C6—C7	−174.0 (2)	C7—C8—C13—N1	−3.1 (3)
C2—C1—C6—C5	0.8 (3)	C9—C8—C13—C12	−0.6 (3)
C2—C1—C6—C7	175.0 (2)	C7—C8—C13—C12	178.3 (2)
C5—C6—C7—O1	122.8 (3)	C9—C10—C14—O2	−164.4 (2)
C1—C6—C7—O1	−51.2 (3)	C11—C10—C14—O2	11.6 (3)
C5—C6—C7—C8	−56.6 (3)	C9—C10—C14—C15	14.6 (3)
C1—C6—C7—C8	129.3 (2)	C11—C10—C14—C15	−169.40 (18)
O1—C7—C8—C9	169.7 (2)	O2—C14—C15—C16	−132.7 (2)
C6—C7—C8—C9	−11.0 (3)	C10—C14—C15—C16	48.2 (3)
O1—C7—C8—C13	−9.2 (4)	O2—C14—C15—C20	42.3 (3)
C6—C7—C8—C13	170.2 (2)	C10—C14—C15—C20	−136.8 (2)
C13—C8—C9—C10	−0.7 (3)	C20—C15—C16—C17	0.3 (3)
C7—C8—C9—C10	−179.60 (19)	C14—C15—C16—C17	175.3 (2)
C8—C9—C10—C11	1.7 (3)	C15—C16—C17—C18	0.3 (4)
C8—C9—C10—C14	177.93 (19)	C16—C17—C18—C19	0.0 (4)
C9—C10—C11—N2	178.84 (19)	C17—C18—C19—C20	−0.9 (4)
C14—C10—C11—N2	2.6 (3)	C18—C19—C20—C15	1.4 (4)
C9—C10—C11—C12	−1.4 (3)	C16—C15—C20—C19	−1.1 (3)
C14—C10—C11—C12	−177.56 (19)	C14—C15—C20—C19	−176.4 (2)

### Hydrogen-bond geometry (Å, °)

**Table:**

Cg1 and Cg2 are the centroids of the rings defined by the atoms C1–C6 and C8–C13, respectively.

**Table:**

*D*—H···*A*	*D*—H	H···*A*	*D*···*A*	*D*—H···*A*
N1—H1B···O1	0.86	2.04	2.676 (3)	130
N2—H2B···O2	0.86	2.05	2.688 (3)	131
C19—H19A···Cg1^i^	0.93	2.79	3.555 (3)	140
N1—H1A···Cg2^ii^	0.86	2.71	3.550 (2)	167

Symmetry codes: (i) *x*−1/2, −*y*−1/2, *z*−1/2; (ii) −*x*+1/2, *y*+1/2, −*z*+1/2.
